# Effect of Bee Venom Acupuncture on Oxaliplatin-Induced Cold Allodynia in Rats

**DOI:** 10.1155/2013/369324

**Published:** 2013-08-22

**Authors:** Bong-Soo Lim, Hak Jin Moon, Dong Xing Li, Munsoo Gil, Joon Ki Min, Giseog Lee, Hyunsu Bae, Sun Kwang Kim, Byung-Il Min

**Affiliations:** ^1^Department of East-West Medicine, Graduate School, Kyung Hee University, Seoul 130-701, Republic of Korea; ^2^Department of Physiology, College of Korean Medicine, Kyung Hee University, Seoul 130-701, Republic of Korea; ^3^Department of Physiology, College of Medicine, Kyung Hee University, Seoul 130-701, Republic of Korea

## Abstract

Oxaliplatin, a chemotherapy drug, often leads to neuropathic cold allodynia after a single administration. Bee venom acupuncture (BVA) has been used in Korea to relieve various pain symptoms and is shown to have a potent antiallodynic effect in nerve-injured rats. We examined whether BVA relieves oxaliplatin-induced cold allodynia and which endogenous analgesic system is implicated. The cold allodynia induced by an oxaliplatin injection (6 mg/kg, i.p.) was evaluated by immersing the rat's tail into cold water (4°C) and measuring the withdrawal latency. BVA (1.0 mg/kg, s.c.) at Yaoyangguan (GV3), Quchi (LI11), or Zusanli (ST36) acupoints significantly reduced cold allodynia with the longest effect being shown in the GV3 group. Conversely, a high dose of BVA (2.5 mg/kg) at GV3 did not show a significant antiallodynic effect. Phentolamine (**α**-adrenergic antagonist, 2 mg/kg, i.p.) partially blocked the relieving effect of BVA on allodynia, whereas naloxone (opioid antagonist, 2 mg/kg, i.p.) did not. We further confirmed that an intrathecal administration of idazoxan (**α**_2_-adrenergic antagonist, 50 **μ**g) blocked the BVA-induced anti-allodynic effect. These results indicate that BVA alleviates oxaliplatin-induced cold allodynia in rats, at least partly, through activation of the noradrenergic system. Thus, BVA might be a potential therapeutic option in oxaliplatin-induced neuropathy.

## 1. Introduction

Colorectal cancer (CRC) was the third most common cancer in both men and women, and it caused about 608,000 deaths in 2008 worldwide, making it the fourth most common cause of death from cancer [1]. Oxaliplatin is an important chemotherapy drug for the treatment of patients with metastatic CRC [2, 3]. Because of its platinum-based molecular structure, it causes a neurotoxic side effect, characterized by the rapid onset of spontaneous severe pain and cold allodynia in the hands, feet, perioral area, or throat, even from a single administration [4, 5]. It is the only major dose-limiting toxicity associated with oxaliplatin use [6]. Since the mechanism is still unclear, an effective treatment of established neuropathic allodynia has yet to be found [7]. Therefore, it would be highly important to find the potential therapeutic options to manage oxaliplatin-induced neuropathic pain.

Bee venom acupuncture (BVA), a treatment method of injecting bee venom (BV) into one or more acupoints, has been used traditionally in East Asia, especially Korea, to relieve pain and treat various diseases, such as arthritis, rheumatism, back pain, sprain, and herniation of nucleus pulposus [8–12]. It is well known that the analgesic effects of acupuncture or electroacupuncture (EA) are mediated by the endogenous opioid and/or noradrenergic system [13–18]. In contrast, the antinociceptive actions of BVA were reported to be mediated by the noradrenergic system, not by the opioids [19–21]. In peripheral nerve-injured rats, Lee and his colleagues have demonstrated that BVA attenuated mechanical allodynia, heat hyperalgesia, and cold allodynia, mainly through the activation of the central noradrenergic system [22–24]. However, the effect of BVA on oxaliplatin-induced neuropathic pain and its mechanism has not been studied. 

In the present study, we investigated whether BVA relieves oxaliplatin-induced cold allodynia, and if so, which endogenous analgesic system is implicated. We report here that BVA has a potent antiallodynic effect on oxaliplatin-induced peripheral neuropathy in rats, which is acupoint- and dose-related, and that it involves the noradrenergic system partially, but not the endogenous opioid system. 

## 2. Materials and Methods

### 2.1. Animals

Adult male Sprague-Dawley rats (210–250 g, 8 weeks old) (Daehan Biolink, Chungbuk, Korea) were housed in cages (3-4 rats per cage) with water and food available *ad libitum*. The room was maintained with a 12 h-light/dark cycle (a light cycle; 08:00–20:00, a dark cycle; 20:00–08:00) and kept at 23 ± 2°C. All animals were acclimated in their cages for 1 week prior to any experiments. All procedures involving animals were approved by the Institutional Animal Care and Use Committee of Kyung Hee University (KHUASP(SE)-12-044) and were conducted in accordance with the guidelines of the International Association for the Study of Pain [25].

### 2.2. Oxaliplatin Injection

As described previously [5, 26], oxaliplatin (Sigma Chemical Co., USA) was dissolved in a 5% glucose (Sigma, USA) solution at a concentration of 2 mg/mL and was intraperitoneally administered at 6 mg/kg. The same volume of 5% glucose solution was injected in the vehicle control group.

### 2.3. Behavioral Tests

To estimate whether cold allodynia was induced, cold immersion test was carried out as described previously [27, 28]. Briefly, each animal was lightly immobilized in a plastic holder and its tail was drooped for proper application of cold water stimuli. The rats were adapted to the holder for 2 days before starting behavioral tests. The tail was immersed in 4°C water, and then the tail withdrawal latency (TWL) was measured with a cut-off time of 15 seconds. The cold immersion test was repeated five times at 5 min intervals. When calculating the average latency, the cut-off time was assigned to the normal responses. The average latency was taken as a measure for the severity of cold allodynia; a shorter TWL was interpreted as more severe allodynia.

Because our previous study showed that a significant allodynic behavior is induced from 3 days after a single oxaliplatin (6 mg/kg, i.p.) injection and lasted up to 1 week after an injection (unpublished data), we tested whether and how BVA relieves oxaliplatin-induced cold allodynia from 3 to 7 days after an oxaliplatin administration.

### 2.4. BVA Treatment

**Figure 1:**
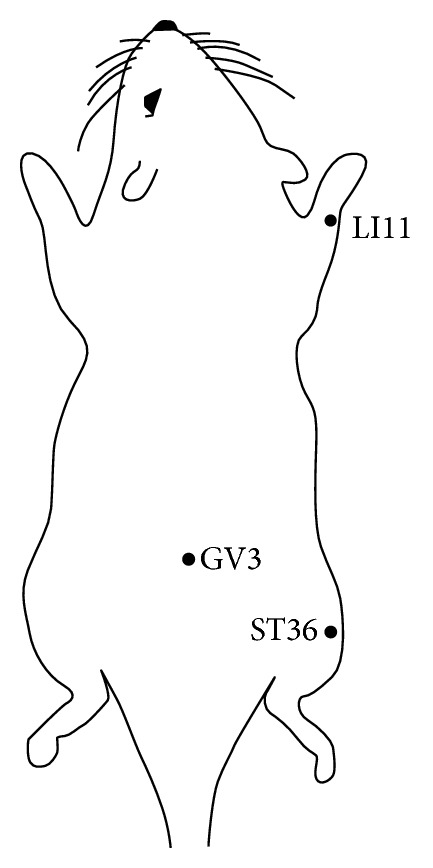
Schematic representation of acupoints used in this study: Quchi (LI11), Zusanli (ST36), and Yaoyangguan (GV3).

To determine the optimal acupoint of BVA, oxaliplatin-injected rats were divided randomly into three groups: Quchi (LI11), Zusanli (ST36), and Yaoyangguan (GV3) groups (*n* = 4/group). After baseline cold sensitivity was measured, BV (1.0 mg/kg) dissolved in normal saline (N/S, 0.05 cc) was injected subcutaneously at GV3, right LI11, or right ST36 acupoints, respectively (Figure 1). The cold immersion test was performed again at 1 hour and 2 hours after BVA. LI11 is located at the depression medial to the extensor carpi radialis, at the lateral end of cubital crease [29]. ST36 is located in the anterior tibial muscle, 5 mm lateral and distal from the anterior tubercle of the tibia [30]. GV3 acupoint is located between the spinous processes of the fourth and the fifth lumbar vertebrae [29].

 In order to find the effective dose of BVA, the rats with cold allodynia were divided randomly into four groups BV2.5, BV1.0, BV0.25, and CON groups (*n* = 4/group). BV (2.5 mg/kg, 1.0 mg/kg or 0.25 mg/kg) dissolved in N/S was injected subcutaneously at GV3 acupoint. To the control group (CON), only 0.05 cc of N/S was injected subcutaneously at the same acupoint. 

### 2.5. Drug Treatment

To investigate the mechanism of BVA, oxaliplatin-injected rats were divided randomly into three groups: N/S + BV, Naloxone + BV, and Phentolamine + BV (*n* = 6/group). After baseline, cold sensitivity was checked; Naloxone + BV and Phentolamine + BV groups were treated intraperitoneally with naloxone and phentolamine (2 mg/kg, dissolved in normal saline to a concentration of 1 mg/mL), respectively. N/S + BV group was treated intraperitoneally with normal saline. Twenty minutes later, all groups were treated subcutaneously with 0.25 mg/kg of BV at GV3 acupoint. To further confirm the noradrenergic mechanism of BVA-induced antiallodynia, an idazoxan (*α*_2_-adrenergic receptor antagonist, 50 *μ*g dissolved in 50 *μ*L N/S) or N/S, was administered intrathecally under isoflurane anesthesia as described previously [31]. The cold immersion test was performed again 30 min after BVA. All drugs were obtained from Sigma-Aldrich (St. Louis, MO, USA).

### 2.6. Statistical Analysis

All the data are presented as mean ± SEM. Statistical analysis and graphic works were done with Prism 5.0 (Graph Pad Software, USA). Paired *t*-test or repeated measures analysis of variance (ANOVA) followed by Dunnett's post hoc test was used for statistical analysis. In all cases, *P* < 0.05 was considered significant. 

## 3. Results

### 3.1. Effects of BVA on Oxaliplatin-Induced Cold Allodynia: Acupoints and Doses

**Figure 2:**
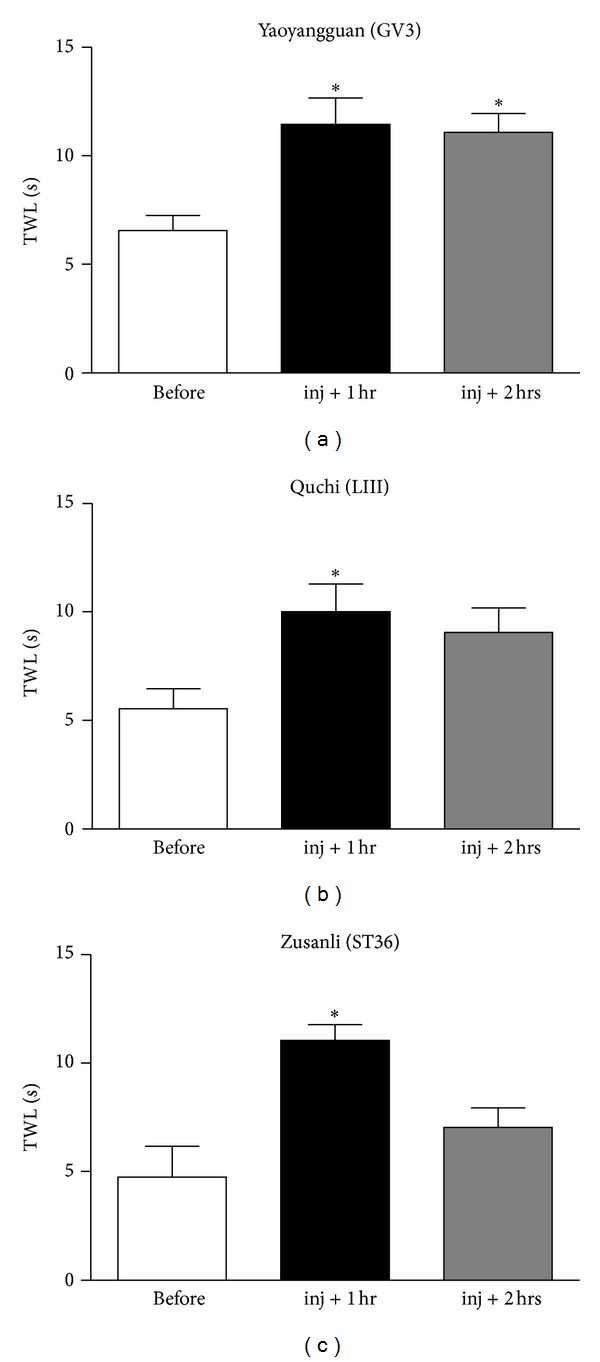
Effects of BVA at different acupoints on oxaliplatin-induced cold allodynia. The behavioral tests for cold allodynia were performed before and after BVA (1.0 mg/kg) at GV3, (a), right LI11 (b), or right ST36 (c) acupoints (*n* = 4/group). In all groups, the tail withdrawal latency (TWL) 1 hr after BVA increased significantly as compared with the TWL before injection. Only GV3 group showed a significant increase in the TWL 2 hr after BVA. Data are presented as mean ± SEM. *P < 0.05 by repeated measures one-way ANOVA followed by Dunnett's post hoc test.

**Figure 3:**
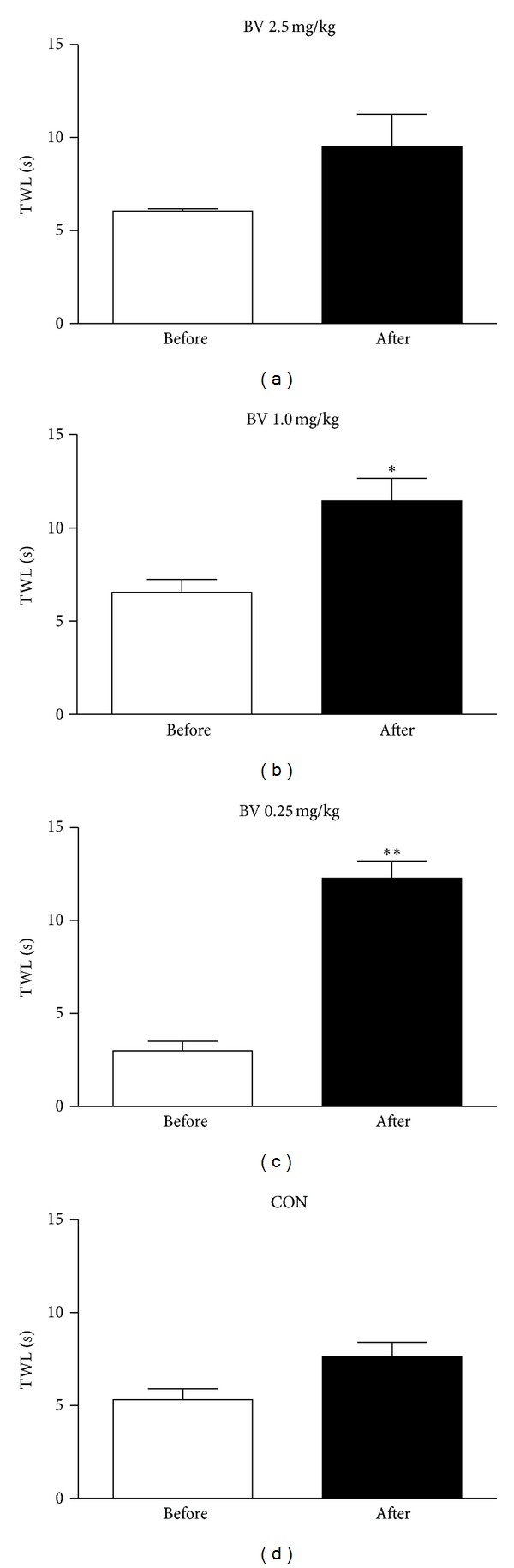
Effects of BVA in different doses of BV on oxaliplatin-induced cold allodynia. The behavioral tests for cold allodynia were performed before and after 2.5 mg/kg (a), 1.0 mg/kg (b), or 0.25 mg/kg (c) BVA treatment at GV3 acupoint (*n* = 4/group). In the control group (CON), only 0.05 cc of normal saline was injected (d). In BV1.0 and BV0.25 groups, the TWL after BVA significantly increased as compared with TWL before BVA. No significant difference between TWLs before and after injection was observed in BV2.5 and CON groups. Data are presented as mean ± SEM. *P < 0.05, **P < 0.01 by paired *t*-test.

The anti-allodynic effects of BVA at different acupoints (GV3, LI11, or ST36) in oxaliplatin-injected rats are shown in Figure 2. In all groups, BVA treatments (1.0 mg/kg, s.c.) significantly increased TWL at 1 hr after BVA as compared with the baseline TWL (*P* < 0.05). Such anti-allodynic effect of BVA at GV3 lasted up to 2 hr after the treatment (*P* < 0.05), whereas the well-known analgesic acupoints, LI11 and ST36 BVA treatments, showed no significant effects at 2 hr after BVA (*P* > 0.05). In control experiments, no significant difference in TWLs before and after a light immobilization without BVA (data not shown) or N/S injection at GV3 (*P* > 0.05, Figure 3(d)) was observed. These results suggest that BVA treatments has potent analgesic actions, of which efficacy is dependent on acupoint. 

Figure 3 shows the effects of BVA with different doses on oxaliplatin-induced cold allodynia. A high dose of BVA (2.5 mg/kg) or N/S injection at GV3 did not show a significant anti-allodynic effect (*P* > 0.05), whereas low doses of BVA (0.25 mg/kg and 1.0 mg/kg) at GV3 markedly increased TWLs 1 hr after injection as compared with the TWL before injection (*P* < 0.01 and *P* < 0.05, resp.). BVA at ST36 or LI11 with the highest (2.5 mg/kg) or lowest (0.25 mg/kg) dose showed no significant anti-allodynic effects (TWLs before versus after BVA: 3.13 ± 1.10 versus 7.35 ± 2.09 (2.5 mg/kg BVA at ST36); 4.65 ± 1.16 versus 8.61 ± 1.25 (2.5 mg/kg BVA at LI11); 4.90 ± 0.75 versus 9.04 ± 2.00 (0.25 mg/kg BVA at ST36); 3.87 ± 0.60 versus. 6.49 ± 2.41 (0.25 mg/kg BVA at LI11); *n* = 4/group, *P* > 0.05 by paired *t*-test).

### 3.2. Effects of Opioid and Adrenergic Receptor Antagonists on BVA-Induced Antiallodynia

**Figure 4:**
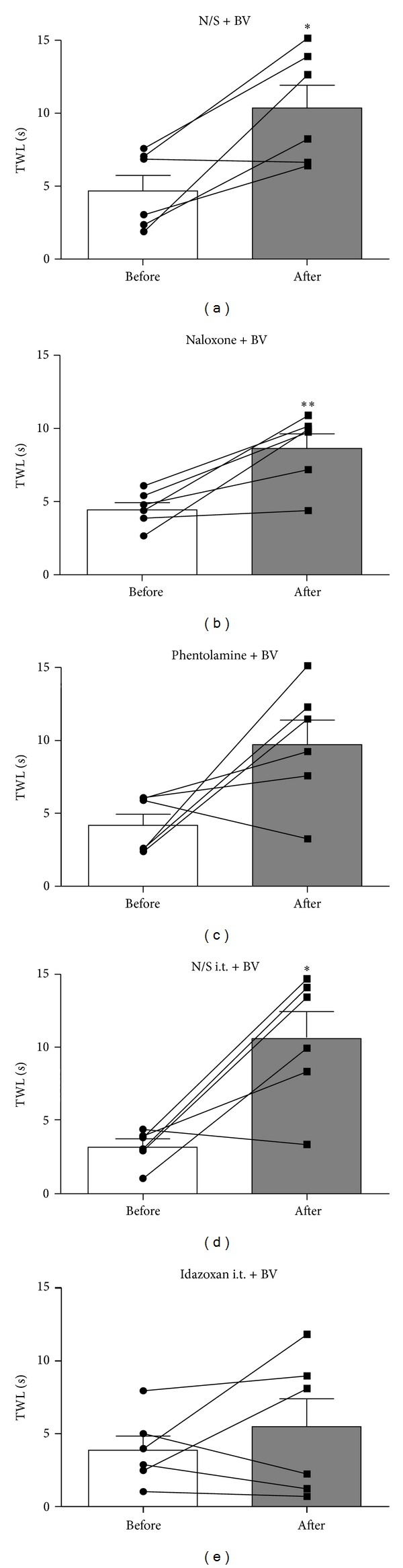
Effects of opioid and adrenergic receptor antagonists on BVA-induced anti-allodynic action. The behavioral tests for cold allodynia were performed before pretreatment of antagonists and after BVA (0.25 mg/kg) treatment at GV3 acupoint. (a) N/S + BV (normal saline (i.p.) pretreatment + BVA, *n* = 6). (b) Naloxone + BV (naloxone (2 mg/kg, i.p.) pretreatment + BVA, *n* = 6). (c) Phentolamine + BV (phentolamine (2 mg/kg, i.p.) pretreatment + BVA, *n* = 6). In N/S + BV and Naloxone + BV groups, TWLs after BVA significantly increased as compared with baseline TWL. In contrast, Phentolamine + BV group showed no significant increase in TWL after BVA. Note that TWL of three subjects of Phentolamine + BV group substantially increased after BVA, whereas TWL of the other three subjects did not. (d) N/S i.t. + BV (normal saline (i.t.) pretreatment + BVA, *n* = 6). (e) Idazoxan i.t. + BV (idazoxan pretreatment (50 *μ*g, i.t.) + BVA, *n* = 6). Data are presented as mean ± SEM. Dots and lines represent the TWL change of individual subject. *P < 0.05, **P < 0.01 by paired *t*-test.

Since 0.25 mg/kg of BVA was slightly more effective than 1.0 mg/kg BVA (Figures 3(b)-3(c)), 0.25 mg/kg of BVA at GV3 was used to see which endogenous analgesic system mediates BVA-induced anti-allodynic action. As shown in Figure 4, phentolamine- (*α*-adrenergic receptor antagonist, 2 mg/kg, i.p.) pretreated group exhibited no significant difference in TWL before and after BVA (*P* > 0.05), suggesting that the noradrenergic system plays a role in mediating the suppressive effect of BVA on oxaliplatin-induced cold allodynia. We further confirmed that an intrathecal (i.t.) injection of idazoxan (*α*_2_-adrenergic receptor antagonist, 50 *μ*g), but not N/S, blocked the BVA-induced anti-allodynic effect (Figures 4(d)-4(e). In contrast, naloxone (opioid antagonist, 2 mg/kg, i.p.) and N/S showed significant increase in TWL after BVA treatment (*P* < 0.01 and *P* < 0.05, resp.), indicating that the endogenous opioid system is not involved in BVA-induced anti-allodynia.

## 4. Discussion

Oxaliplatin is a platinum-based third-generation chemotherapy drug to treat patients with metastatic CRC [2, 3]. Thus, it is structurally similar to cisplatin and carboplatin and has a neurotoxic side effect, but no nephrotoxicity and hematotoxicity have been observed [2, 4]. This acute oxaliplatin-induced neurotoxicity is developed even through a single oxaliplatin administration [5, 26]. There were only a few reports showing the effective treatment or prevention of oxaliplatin-induced neuropathic pain symptoms. Although the intravenous calcium and magnesium therapy could attenuate the development of oxaliplatin-induced neuropathy [32, 33], it was not complete yet in the treatment of the established allodynia [7, 34]. Therefore, it is now required to find the potential therapeutic options to manage oxaliplatin-induced neuropathic pain.

This study clearly shows that BVA has a potent anti-allodynic effect in oxaliplatin-injected rats (Figures 2 and 3). To see acupoint-dependent effect, we examined which acupoint has the most relieving effect on oxaliplatin-induced cold allodynia. Although BVA at LI11, ST36, and GV3 acupoints all had significant analgesic effects, BVA at GV3 acupoint had a longer lasting effect than the other acupoints (Figure 2). This duration of anti-allodynic action of BVA at GV3 (lasting for ~2 hr) is clinically important because morphine (1, 2, and 4 mg/kg, i.p.) showed no significant analgesic effect at 2 hr after its administration [26]. It also should be noted that GV3 acupoint is closer to the tail, where cold immersion test was performed, than the other acupoints. Interestingly, we found that EA stimulation at a point on the hind limb had a greater anti-allodynic effect than EA at a point that is close to the tested tail (unpublished data). These results might suggest that proximal acupoint stimulation is more effective in BVA treatment on oxaliplatin-induced neuropathic pain, whereas distal acupoint stimulation is more effective in EA treatment. 

In regard to the effective dose of BVA, we demonstrate that a low dose of BVA (1.0 or 0.25 mg/kg) has a significant anti-allodynic effect in oxaliplatin-injected rats while a high dose of BVA (2.5 mg/kg) has no significant effect (Figure 3). Such result is different from the results in a previous study by Kang et al. [23] showing that a low dose (0.25 mg/kg) of BVA did not produce significant anti-allodynic effect, while a high dose of BVA (2.5 mg/kg) significantly reduced cold allodynia in a rat model of sciatic nerve chronic constriction injury (CCI). This discrepancy might be due to the differences in pain model (sciatic nerve CCI versus oxaliplatin), the region of the cold allodynia test (hind paw versus tail), and acupoints used (ST36 versus GV3). In the present study, however, one of the rats treated with a high dose (2.5 mg/kg) of BVA showed a gradual decrease in TWL (before BVA = 6.40 sec; 1 hour after BVA = 4.89 sec; 2 hours after BVA = 3.85 sec), suggesting more severe pain. BV is also called a “double-edged sword” having nociceptive and antinociceptive effects together [35]. Therefore, it might be of high importance to find a proper concentration of BVA through disease-by-disease approaches. In ongoing studies, the optimal dose of BVA in various disease models is to be investigated.

Acupuncture has been used for thousands of years in East Asia including Korea, China, and Japan to treat various diseases generating few side effects. In recent years, it has received attention as an alternative method of medicine in Western countries [36, 37]. For decades ago, it has been demonstrated in many clinical and animal studies that acupuncture or EA analgesia is mediated by the endogenous analgesic systems, especially opioid [13, 16, 18, 38] and noradrenergic inhibitory systems [14, 15, 17, 39, 40]. Our previous studies using a rat model of peripheral nerve injury suggested that both of the opioid and noradrenergic systems equally contributed to the anti-allodynic effects of EA [41–43]. On the other hand, BVA has been reported to attenuate neuropathic pain symptoms induced by CCI through activation of *α*_2_-adrenergic receptors, but not opioid receptors, in the rat spinal cord [22–24]. Similarly, the present results (Figure 4) suggest that the relieving effect of BVA on oxaliplatin-induced cold allodynia involves the noradrenergic, but not opioid, system. Thus, BVA and EA might have different mechanisms of anti-allodynic action in oxaliplatin-induced neuropathy. However, the anti-allodynic effect of BVA in this study was just partially blocked by phentolamine pretreatment (*P* = 0.065). The TWL of three subjects of Phentolamine + BV group substantially increased after BVA as compared with baseline TWL, whereas the TWL of the other three subjects did not. This might be due to individual differences in response to phentolamine, as previously shown in nerve injury models [44]. Alternatively, the other descending inhibitory systems like serotonergic, GABA, and/or cholinergic systems might be involved [19, 30, 43]. Further studies on this issue may increase our understanding of the neurological mechanisms of BVA analgesia.

## 5. Conclusions

In conclusion, our findings in the present study suggest that BVA has a potent relieving effect on oxaliplatin-induced cold allodynia and that GV3 acupoint and low dose of BV have an optimal effect. Such anti-allodynic effect of BVA is partially mediated by the noradrenergic, but not opioid, system. Thus, we propose that BVA treatment can be a potential therapeutic option in oxaliplatin-induced neuropathic pain.
